# Urinary glutamine/glutamate ratio as a potential biomarker of pediatric chronic intestinal pseudo-obstruction

**DOI:** 10.1186/s13023-017-0615-3

**Published:** 2017-03-28

**Authors:** Jun-Kai Yan, Ke-Jun Zhou, Jian-Hu Huang, Qing-Qing Wu, Tian Zhang, Chao-Chen Wang, Wei Cai

**Affiliations:** 1 Department of Pediatric Surgery, Xin Hua Hospital Affiliated to Shanghai Jiao Tong University School of Medicine, 1665 Kongjiang Rd, Shanghai, 200092 China; 2 Shanghai Institute for Pediatric Research, Shanghai, China; 3 Shanghai Key Laboratory of Pediatric Gastroenterology and Nutrition, Shanghai, China; 4 Department of Public Health, Aichi Medical University, Aichi, Japan

**Keywords:** Chronic intestinal pseudo-obstruction, Short bowel syndrome, Urinary glutamine/glutamate ratios, Biomarker

## Abstract

Chronic intestinal pseudo-obstruction (CIPO) is a rare intestinal motility disorder with significant morbidity and mortality in pediatric patients. The diagnosis of CIPO is difficult, because it is clinically based on the symptoms and signs of bowel obstruction which are similar to the clinical manifestations of other gastrointestinal diseases like short bowel syndrome (SBS). Therefore, it is desirable to identify and establish new laboratory diagnostic markers for CIPO that are reliable and easily accessible. In our study we have identified the ratio of the urinary glutamine and glutamic acid as a promising biomarker for distinguishing suspected CIPO cases and simple SBS cases. The area under ROC curve was 0.83, at cutoff value = 7.04 with sensitivity of 65% and specificity of 92%.

**Electronic supplementary material:**

The online version of this article (doi:10.1186/s13023-017-0615-3) contains supplementary material, which is available to authorized users.

Dear Editor,

Chronic intestinal pseudo-obstruction (CIPO) is a rare disorder of intestinal motility characterized by severe and disabling repetitive episodes or continuous symptoms and signs of bowel obstruction, in the absence of a fixed, lumen-occluding lesion. Its prevalence is about 1 in 40,000 to 100,000 live births [1, 2], and the overall mortality rate has been reported to be between 10 and 32% [3]. The quality of life for patients with CIPO is poor due to persistent symptoms, frequent emergency room visits and hospitalizations, and the need for nutrition support. A large proportion of patients become malnourished with up to one-third of adults and 80% of children requiring long-term home parenteral nutrition. The clinical picture tends to be dominated by abdominal distention (98%), vomiting (91%; bilious in 80%), abdominal pain (58–70%), failure to thrive (62%), diarrhea (31–42%), constipation (42–77%), feeding intolerance (39%), and urinary symptoms (11%), which are particularly severe during episodes of pseudo-obstruction [4]. Notably, pseudo-obstruction is a term used to define a heterogeneous group of neuromuscular disorders, which can be further classified into three major types: neuropathies, myopathies and mesenchymopathies, based on the histological underlying abnormalities of enteric neurons, smooth muscle cells and interstitial cells of Cajal (ICC), respectively. However, no matter the etiology, the end result presents markedly compromised peristalsis within the gastrointestinal (GI) tract. Thus, CIPO should be considered as a description of symptoms rather than a true disease.

The first challenge for physicians dealing with these patients is to establish a firm diagnosis from those above-mentioned symptoms, which are nonspecific and similar to the clinical manifestations of other gastrointestinal diseases like short bowel syndrome (SBS). Misdiagnosis or a delay in diagnosis typically result in the disease going unrecognized for long periods, which means that patients often undergo repeated and potentially dangerous diagnostic tests and treatments. Currently, a stepwise approach has been used to make the diagnosis of CIPO, including pertinent laboratory studies, plain films of the abdomen, GI transit measurements, and specialized tests of GI motility [5]. However, the diagnosis of CIPO can be elusive for a number of reasons: 1) symptoms typically evolve slowly over a number of years rather than develop at once; 2) initial diagnostic tests (ie, endoscopy and abdominal ultrasound) are usually normal; 3) Biologic markers for CIPO are not available. Therefore, it is desirable to identify and establish new laboratory diagnostic markers for CIPO that are reliable and easily accessible.

By liquid chromatography/mass spectrometry (LC/MS)-based urinary amino acid metabolomic profiling, we have identified the ratio of the urinary glutamine (Gln) and glutamate (Glu) as a promising biomarker for aiding distinguish suspected CIPO cases and simple SBS cases. Here, SBS patients were enrolled for the following reasons: 1) Up to 80% of the pediatric patients who develop symptoms suggestive of intestinal failure (IF) are SBS patients in our department. Therefore, in order to effectively screen out potential CIPO patients from the whole group of IF patients, SBS patients would be an appropriate control group because of its good representativeness. 2) Normally, SBS patients do not have obstruction, however, those with poor enteral feeding tolerance may have such obstruction-like symptoms as abdominal distention, vomiting and abdominal pain, especially after receiving inappropriate enteral nutrition. On the other hand, severe cases may exist who combine short bowel with dysmotility. In addition to screening out potential CIPO patients, our results may also aid distinguish whether the obstruction-like symptoms in SBS patients are attributed to dysmotility or simply enteral feeding intolerance.

In the current study, though the exact mechanism needs to be further investigated, here, we propose an “energy-based acidosis” hypothesis. Given that urinary excretion of glutamine and glutamate is sensitive to environmental pH, renal catabolism of glutamine is accelerated during chronic metabolic acidosis, leading to increased excretion of glutamate and decreased excretion of glutamine. As a result, the ratio of the urinary glutamine and glutamate is decreased, compared to the normal [6]. We hypothesized that patients with CIPO may represent chronic acidosis due to the compromised energy metabolism in intestinal smooth muscle cells, in which decreased aerobic oxidation along with increased anaerobic glycolysis may lead to the accumulation of acidic metabolites. These acidic metabolites may chronically affect the overall body condition, and therefore, for CIPO patients, this ratio not only reflects the severity of acidosis, but also substantially reflects the severity of compromised energy metabolism within the GI tract.

Since SBS patients do not represent severe disorders of energy metabolism while CIPO patients do, we hypothesize that the urinary Gln/Glu ratio may serve as a potential diagnostic biomarker for CIPO.

## Methodology

This study was approved by the Ethics Committee of Xin Hua Hospital. Written informed consent for sample collection was obtained from the patients’ parents or guardians. To test whether urinary Gln/Glu ratio may serve as a potential diagnostic biomarker for CIPO, we determined urinary Gln and Glu by LC/MS in 197 spot urine samples, including 102 samples from 8 clinically defined CIPO patients, 53 samples from 10 SBS patients and 42 samples from 42 healthy controls. The study cohort included: clinically defined CIPO patients (*n* = 8; aged 0.8–9.2 years, median age = 4.9 years); SBS patients (*n* = 10, aged 1.1–13.1 years, median age = 5.8 years) and healthy controls (*n* = 42, aged 5–12 years, median age =6.7 years). Median remnant bowel length in SBS group was 53.0 cm (23–90 cm). Spot urine samples were collected three times a week, at 8:00 a.m and 8:00 p.m.

## Findings

**Fig. 1:**
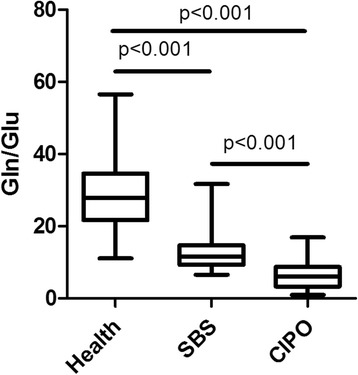
Box-and-whisker plot showing urinary Gln/Glu ratios. Urinary Gln/Glu ratios in the healthy controls, SBS patients and CIPO patients are 28.7 ± 10.8 (*n* = 42), 13.3 ± 6.3 (*n* = 53 samples from 10 patients) and 6.5 ± 4.0 (*n* = 102 samples from 8 patients), respectively

**Fig. 2:**
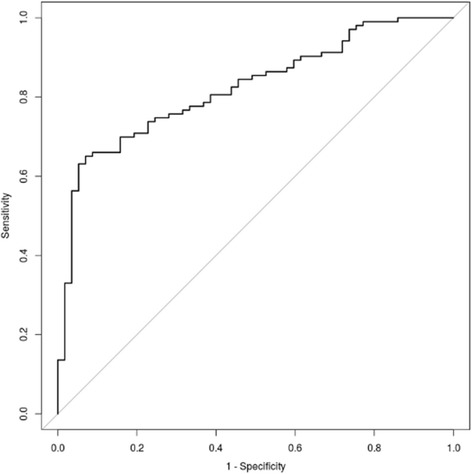
The ROC curve for Gln/Glu ratios to differentiate CIPO from simple SBS. The area under ROC curve was 0.83, at cutoff value = 7.04 with sensitivity of 65% and specificity of 92%

Urinary Gln/Glu ratios are shown in Fig. 1. Compared to control Gln/Glu ratios (28.7 ± 10.8, *n* = 42), we found markedly decreased Gln/Glu ratios in SBS patients (13.3 ± 6.3, *n* = 53 samples from 10 patients, *p* < 0.001) and in CIPO patients (6.5 ± 4.0, *n* = 102 samples from 8 patients, *p* < 0.001). Also note that Gln/Glu ratios in CIPO patients were significantly lower than those in SBS patients (*p* < 0.001). The ROC curve for Gln/Glu ratios to differentiate CIPO from simple SBS cases is shown in Fig. 2. Data was analyzed by using 155 samples from 8 CIPO patients and 10 SBS patients. The area under ROC curve was 0.83, at cutoff value = 7.04 with sensitivity of 65% and specificity of 92%. As part of the raw data, absolute concentrations of Gln and Glu, as well as Gln/Glu ratios in 3 representative patients are shown in Additional file 1.

## Conclusions

These results identify the urinary Gln/Glu ratio as a promising biomarker for CIPO. In particular, it has the potential to differentiate suspected CIPO from simple SBS cases, and may improve the efficacy of the final diagnosis. The optimal relationship between sensitivity and specificity for minimum urinary Gln/Glu ratio in differentiating CIPO and SBS was 7.04, which means patients whose urinary Gln/Glu ratio <7.04 should be considered as suspected CIPO. However, a validation cohort with more samples will be needed to validate these findings. Therefore, with this report we hope to attract more CIPO cases in order to statistically validate our preliminary study.

## Additional file

Additional file 1: Absolute concentrations of Gln and Glu, as well as Gln/Glu ratios in 3 representative patients. (DOCX 214 kb)

## Abbreviations

CIPO: Chronic intestinal pseudo-obstruction
GI: Gastrointestine
Gln: Glutamine
Glu: Glutamate
ICC: Interstitial cells of Cajal
LC/MS: Liquid chromatography/mass spectrometry
SBS: Short bowel syndrome
