# Supplementary material for: Urinary glutamine/glutamate ratio as a potential biomarker of pediatric chronic intestinal pseudo-obstruction
Source: Orphanet J Rare Dis. 2017 Mar 28;12:62. doi: 10.1186/s13023-017-0615-3 (PMC5371254; doi:10.1186/s13023-017-0615-3)
Supplement: Additional file 1: — Absolute concentrations of Gln and Glu, as well as Gln/Glu ratios in 3 representative patients. (DOCX 214 kb) [file 13023_2017_615_MOESM1_ESM.docx]

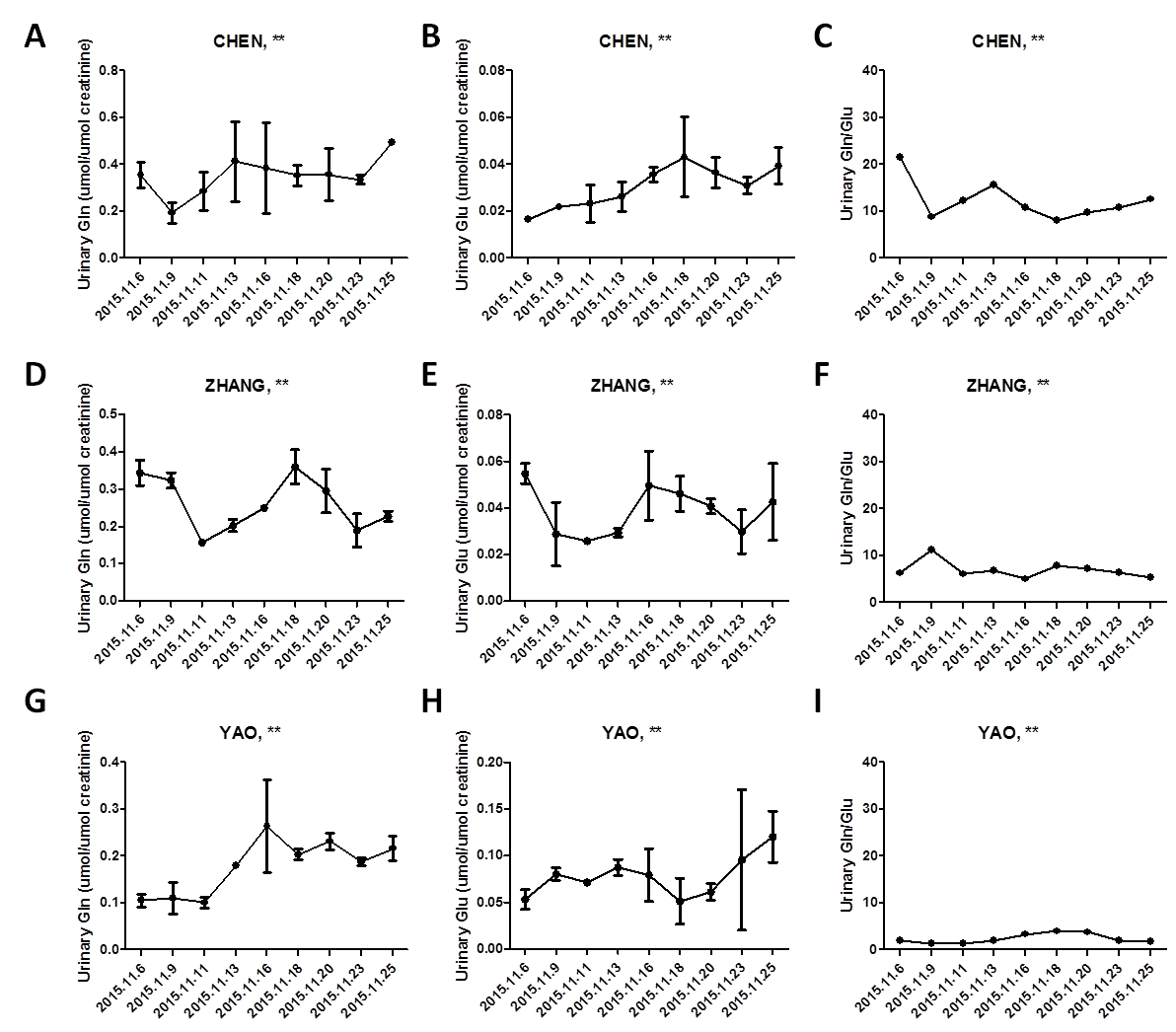


Supplementary data for review. Absolute concentrations of Gln and Glu, as well as Gln/Glu ratios in 3 representative patients. A-C: CHEN was a female patient with SBS, 1 year old, and her Gln/Glu ratios remained almost stable at roughly 15 within 3 weeks. D-F: ZHANG was a female patient with CIPO, 1 year old, and her Gln/Glu ratios remained almost stable at roughly 8 within 3 weeks. G-I: YAO was a male patient with CIPO, 9 years old, and his Gln/Glu ratios remained almost stable at roughly 2 within 3 weeks.
